# Expression of BMI-1 and Mel-18 in breast tissue - a diagnostic marker in patients with breast cancer

**DOI:** 10.1186/1471-2407-10-686

**Published:** 2010-12-16

**Authors:** Margit LH Riis, Torben Lüders, Anne-Jorunn Nesbakken, Hilde S Vollan, Vessela Kristensen, Ida RK Bukholm

**Affiliations:** 1 Department of Surgery, Akershus University Hospital, Lørenskog, Norway; 2 Department of Clinical Molecular Biology (EpiGen), Institute of Clinical Medicine, Akershus University Hospital, University of Oslo, Lørenskog, Norway; 3 Department of Pathology, Akershus University Hospital, Lørenskog, Norway; 4 Institute of Clinical Medicine, University of Oslo, Lørenskog, Norway

## Abstract

**Background:**

Polycomb Group (PcG) proteins are epigenetic silencers involved in maintaining cellular identity, and their deregulation can result in cancer. Expression of Mel-18 and Bmi-1 has been studied in tumor tissue, but not in adjacent non-cancerous breast epithelium. Our study compares the expression of the two genes in normal breast epithelium of cancer patients and relates it to the level of expression in the corresponding tumors as well as in breast epithelium of healthy women.

**Methods:**

A total of 79 tumors, of which 71 malignant tumors of the breast, 6 fibroadenomas, and 2 DCIS were studied and compared to the reduction mammoplastic specimens of 11 healthy women. In addition there was available adjacent cancer free tissue for 23 of the malignant tumors. The tissue samples were stored in RNAlater, RNA was isolated to create expression microarray profile. These two genes were then studied more closely first on mRNA transcription level by microarrays (Agilent 44 K) and quantitative RT-PCR (TaqMan) and then on protein expression level using immunohistochemistry.

**Results:**

Bmi-1 mRNA is significantly up-regulated in adjacent normal breast tissue in breast cancer patients compared to normal breast tissue from noncancerous patients. Conversely, mRNA transcription level of Mel-18 is lower in normal breast from patients operated for breast cancer compared to breast tissue from mammoplasty. When protein expression of these two genes was evaluated, we observed that most of the epithelial cells were positive for Bmi-1 in both groups of tissue samples, although the expression intensity was stronger in normal tissue from cancer patients compared to mammoplasty tissue samples. Protein expression of Mel-18 showed inversely stronger intensity in tissue samples from mammoplasty compared to normal breast tissue from patients operated for breast cancer.

**Conclusion:**

Bmi-1 mRNA level is consistently increased and Mel-18 mRNA level is consistently decreased in adjacent normal breast tissue of cancer patients as compared to normal breast tissue in women having had reduction mammoplasties. Bmi-1/Mel-18 ratio can be potentially used as a tool for stratifying women at risk of developing malignancy.

## Background

Breast cancer is the leading cause of cancer mortality in women [1]. The prognosis of breast cancer is dependent on stage at the diagnosis, tumors diagnosed at early stage having better prognosis. It is therefore important to detect breast tumors at as early stage as possible [2,3]. Benign diseases, like fibroadenomas, in the mammary gland are associated with increased risk of breast cancer in the same women, although in clinical practice it is difficult to recognize women with fibroadenomas who are at risk of developing breast cancer. There is a need for diagnostic tools which may help stratifying the risk for women with benign changes in their breasts. Bmi-1 and Mel-18 may be genes used for this purpose as our study will show.

There is increasing evidence that breast cancers arise from deregulation of normal pathways in stem or early progenitor cells due to mutations or epigenetic silencing [4,5]. Polycomb Group (PcG) proteins are epigenetic silencer genes involved in maintaining cellular identity, and their deregulation can result in cancer [5]. The deregulation of PcG genes may be one of the first events in neoplasia of breast epithelium. Bmi-1 and Mel-18 are two members of the PcG family. Bmi-1 has been shown to maintain the stem cell pool by preventing premature senescence [6,7]. Bmi-1 expression has been detected in normal mammary epithelium and myoepithelial cells and later studies have implied Bmi-1 in connection with self-renewal of stem cells in breast tissue [8]. Bmi-1 has been shown to be up-regulated in breast tumors [7] as well as in several other tumor types [7,9-12]. Over-expression induces lymphomas [11,13,14]. Bmi-1 has shown to regulate cellular senescence and proliferation in rodent and human fibroblasts [15]. After a finite number of cell division, most human cells undergo cellular senescence, whereby cells irreversibly cease to divide [16,17]. Bmi-1 can also bypass senescence and immortalize human mammary epithelial cells [18]. Bmi-1 has been studied in plasma of breast cancer cells with healthy women as controls and results show that levels of Bmi-1 expression may be a surrogate marker of poor prognosis [19]. This may be a very useful noninvasive prognostic marker.

Mel-18 regulates cell proliferation and senescence via transcriptional repression of Bmi-1 and c-myc oncoproteins [17], and is considered to play a dual role, being either oncogenic in some tumor types or acting as a tumor suppressor gene in others. In breast cancer, Mel-18 is supposed to play a tumor suppressor role [20]. Mel-18 was originally cloned from B16 mouse melanoma cells and was shown to be highly expressed in many tumor cells including human melanoma and Hodgkin's lymphomas [21,22].

It has been of our interest to investigate to what extent normal breast tissue from breast cancer patients is actually normal and to what it reflects cancer-specific deregulation. Since both Mel-18 and Bmi-1 may play a role in renewal of stem cells, deregulation of these proteins may be one of the initial steps in development of neoplasia and may be present even in non-cancerous tissue adjacent to the tumor tissue. In the present study, we wanted to analyze whether expression of these two genes, both at the mRNA and protein level, differ between normal tissue taken from breast cancer patients compared to breast tissue from women who had no actual or previous history of any kind of malignancy in the breast.

## Methods

### Tissue Collection

Tissue samples from malignant tumors and normal counterpart were obtained from patients operated for breast cancer at Akershus University Hospital in the period 2003-2009. In addition to the operated tumor, in some cases large core needle biopsies were taken preoperatively at the radiologist department either when women came for screening or for diagnostic mammography due to palpable mass. Both tumors and biopsies were evaluated by a pathologist to confirm the diagnosis and estimate the tumor cell content. All tumor samples used in this study contained at least 60% tumor cells. Tissue samples were immediately stabilized in RNAlater, and then stored at -80°C. The women have signed a written consent to participate in the study. The study was approved by the Regional Committee for Medical and Health Research Ethics (REK). A total numbers of 71 samples from tumors were analyzed. Mostly invasive ductal carcinomas (64 samples) were included in the study. The remaining were either lobular (5 samples) or mixed (2 samples). Mean age at the operation was 60 years, median 60 years (range 34-83). 30 were classified as lymph node positive and 41 as lymph node negative. 55 were estrogen positive and 43 samples were positive for progesterone. Her-2 gene was found to be amplified in 4 samples (only 21 of the samples were tested for Her-2 gene amplification). Samples from 2 DCIS lesions as well as samples from 6 fibroadenomas were also included in the study. The clinical data are summarized in tables 1 and 2.

**Table 1:** Clinical data for the tumor patients.

Histology	
Ductal	64

Lobular	5

Mixed	2

Palpable	

Yes	54

No	17

Grade	

1	8

2	40

3	23

Lymph node status	

N0	44

N1	19

N2	6

N3	2

Estrogen receptor status	

ER+	56

ER-	15

Progesterone receptor status	

PR+	41

PR-	30

HER2 receptor status	

HER2+	4

HER2-	17

Unknown	50

	

Recurrence/Metastasis	1

Death (not cancer specific)	2

**Table 2:** Age distribution for the patients within the different groups

	Cancers	Non cancer controls	DCIS	Fibroadenomas
**Mean Age**	60	39	63	42

**Median Age**	60	36	63	45

**Range**	34-83	20-68	60-66	26-52

Tissue samples from non cancer controls (reduction mammoplasty) have been gathered since the autumn of 2008 and stabilized in the same way as the other samples. Mean age of these women was 39 years, median 36 years (range 20-68). Only two of these women were postmenopausal. Our intension was to have a cohort of healthy women to compare our results. These women also signed a written consent and their names and identification number were registered in our databases.

### RNA isolation

The surgical specimens and the large core needle biopsies were macroscopically dissected to obtain a sample suitable for further processing. Frozen tissue was homogenized in Trizol (Invitrogen) with a 5 mm steal bead (Qiagen) using a Mixer Mill MM301 (Retsch) at 30 Hz for 2 min. RNA was isolated following the manufacturer's protocol or the protocol of Wei and Kahn [23]. Purified RNA was dissolved in RNase-free water (Ambion). Concentration was measured using NanoDrop and RNA quality assayed on an Agilent 2100 BioAnalyzer. The purified RNA was stored at -80°C.

### Microarray Analysis and Statistical Analysis

500-1000 ng isolated RNA was converted to cDNA with reverse transcriptase and an oligo(dT) primer bearing a T7 promotor followed by in vitro transcription with T7 RNA polymerase to create amplified antisense RNA. The amplified RNA was labeled with Cy3 or Cy5. As a reference probe universal human reference RNA (UHR; Stratagene) was amplified and labeled as above. Amplification and labeling efficiency was controlled using a NanoDrop.

Labeled cRNA was hybridized to Agilent Whole Human Genome Oligo Microarrays per the manufacturer's protocol. After hybridization for 17 hours the arrays were washed and scanned using an Agilent scanner and microarray data extracted with Agilent Feature Extraction software. Preprocessing of the microarray data was done in J-Express Pro http://www.molmine.org while between-array quantile normalization was done in BioConductor [24]. The microarray data are submitted to The ArrayExpress Archive http://www.ebi.ac.uk/microarray-as/ae/ accession number E-MTAB-271.

Further statistical analysis was done in R. Between group comparison were done using Student's two-sided, two-class t-test and ANOVA using the function *aov*.

### Quantitative Real-time PCR (qPCR)

Total RNA was reverse transcribed using the High Capacity RNA to-cDNA Master Mix (Applied Biosystems) and cDNA was diluted with high molecular grade water and stored at -20°C.

For qPCR, 25 ng cDNA, primer/probe sets and TaqMan Gene Expression Master Mix (2×) (Applied Biosystems) were pipetted on a MicroAmp Optical 96-Well Reaction Plate (Applied Biosystems) using the epMotion 5075 pipetting robot (Eppendorf). All samples were pipetted in triplicates and a no template control was run on each plate. The plate was run on the ABI PRISM 7900 HT Fast Real-Time PCR system (Applied Biosystems) with the thermal profile: 50°C for 2 min, 95°C for 10 min and 50 cycles at 95°C for 15 seconds and 60°C for 1 min. Analysis were done using ABI Prism SDS2.3 software and the RQ Manager 1.2 (Applied Biosystems). As for the microarray studies the UHR RNA was used as calibrator.

TaqMan^® ^Gene Expression Assays from Applied Biosystems (BMI1: Hs00180411_m1, PCGF2: Hs00810639_m1, MRPL19: Hs00608519_m1, PPIA: Hs99999904_m1) were used to perform qPCR. All gene assays target exon-exon junctions to be mRNA specific. The final concentration of the TaqMan gene expression assay used was 900 nM for each primer and 250 nM for each probe.

### Histology

The slides were evaluated by an experienced pathologist (AJN) and graded according to the Nottingham grading system (Nottingham modification of the Bloom-Richardson system) [25].

### Immunohistochemistry

Immunohistochemistry was performed on 5 μm sections from formalin-fixed, paraffin-embedded tissue applied to coated slides. Deparaffinization, rehydration and epitope retrieval were performed in a Dako PT link (Dako) at 97°C for 20 minutes. Dako Autostainer Plus together with Envision™ Flex, high pH system (K8000, Dako) were used in the immunostaining procedure following the operating manual. The secondary antibody was incubated for 20 minutes. Sections were stained with anti-Bmi-1 (Santa Cruz Biotechnology) dilution 1:150 and anti-Mel-18 (Santa Cruz Biotechnology) dilution 1:75. Primary antibody incubated for 30 minutes. For Mel-18 visualization a FLEX + Rabbit (Linker) protocol was used. The choice of antibodies was made primarily on the basis of literature studies, choosing clones used in previous publications [20,26]. The slides were counterstained with Hagen's Hematoxylin for visualization of tissue structures.

### Evaluation of immunohistochemistry

The amount of positive cells and immunoreactivity intensity was evaluated semi- quantitative. For Bmi-1 only two grades were applied; if less than 10% of the epithelial cells were immunoreactive to Bmi-1, the sample was recorded as negative for Bmi-1 immunoreactivity, while samples showing more than 10% of cells were recorded as positive.

Soring grades for Mel-18 were as follows: Grade 1; < 5% of the cells positive for Mel-18. Grade 2; 6% to 35% showing positive immunoreactivity. Grade 3; 36-70% of the cells showed positive immunoreactivity, and grade 4 when more than 70% of the cells were positive for Mel-18 immunoreactivity. The intensity of the immunoreactivity was also recorded, and the grading was as follows: Grade 1, weak intensitivity, grade 2; moderate intensitivity and grade 3 when a strong intensitivity of the immunoreaction was observed. The immunoreactivity for both Mel-18 and Bmi-1 was evaluated by two independent investigators. There was no discrepancy between the two investigators.

## Results

### Bmi-1 transcription level

**Figure 1:**
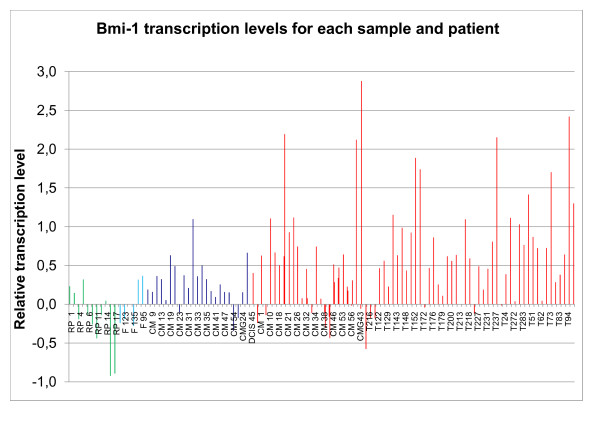
**Bmi-1 transcription levels for each sample and patient**. Green = non cancer controls (NC), light blue = fibroadenomas (F), dark blue = adjacent normal (N), and red = tumor (T). In cases where more than one tumor sample is shown for one patient, both preoperative and peroperative tumor tissue were available. On the y-axis there is a logarithmic transcription level of our sample compared to a Universal Human Reference (log_2_(expression of sample/expression of UHR)).

**Figure 2:**
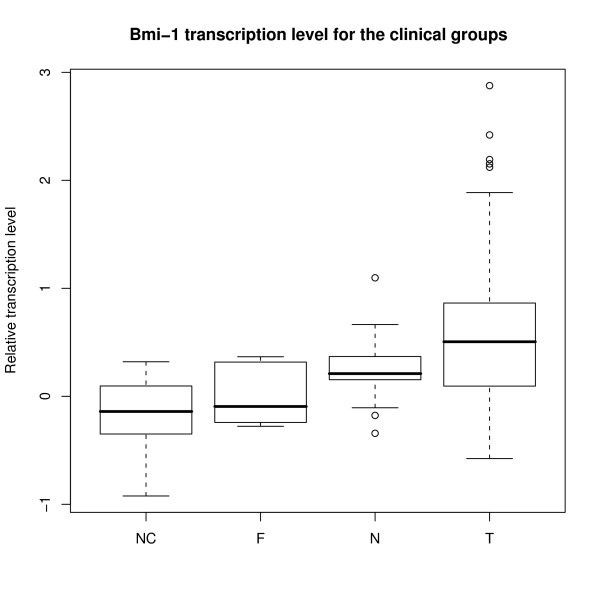
**Bmi-1 transcription levels for the clinical groups**. Boxplot showing the transcription level of Bmi-1 within the clinical groups. Group legends as in figure 1.

When comparing the transcription level of Bmi-1 in the different clinical groups, i.e. breast cancer, tissue taken in the vicinity of the tumor, fibroadenomas and breast tissue from non cancer controls, statistically significant differences were observed between the groups (table 3). Transcription level in non cancer breast controls was lowest, while transcription level in the normal adjacent tissue was more similar to that of the tumor (Figure 1 and 2).

**Table 3:** Mean transcription levels of Bmi-1 and Mel-18 in the different groups as well as the p-values and confidence intervals for the group-wise comparisons.

Bmi-1	Group	mean	SD	p-value (confidence interval)		
				
				F	N	T
	
	NC	0.098	0.184	<0.001 (-0.424 -0.149)	0.0418 (-0.346 -0.007)	<0.001 (-0.790 -0.419)
	
	F	0.385	0.080		0.124 (-0.032 0.253)	<0.001 (-0.480 -0.157)
	
	N	0.274	0.295			<0.001 (-0.620 -0.237)
	
	T	0.703	0.659			
**Mel-18**	**Group**	**mean**	**SD**	**p-value (confidence interval)**		
				
				**F**	**N**	**T**
	
	**NC**	0.929	0.139	<0.001 (0.230 0.503)	<0.001 (0.362 0.707)	<0.001 (0.405 0.706)
	
	**F**	0.563	0.115		0.065 (-0.0115 0.347)	0.021 (0.030 0.348)
	
	**N**	0.395	0.352			0.827 (-0.172 0.214)
	
	**T**	0.373	0.556			

Of special interest was the difference in transcription levels of Bmi-1 mRNA between non cancer controls and normal tissue from cancer patients (p = 0.041).

### Mel-18 transcription level

**Figure 3:**
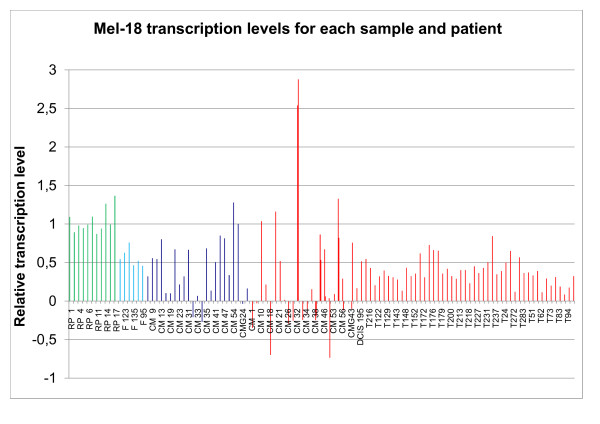
**Mel-18 transcription levels for each sample and patient**. Green = non cancer controls (NC), light blue = fibroadenomas (F), dark blue = adjacent normal (N) and red = tumor (T). In cases where more than one tumor sample is shown for one patient, both preoperative and peroperative tumor tissue were available. As in figure 1 there is logarithmic transcription level on the y-axis.

**Figure 4:**
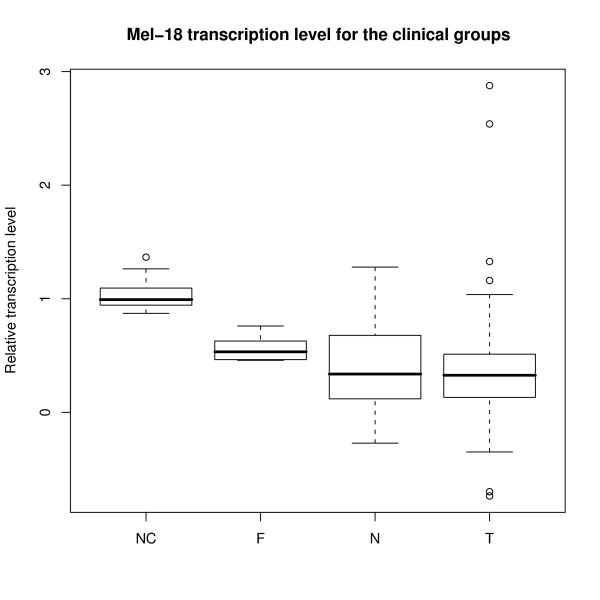
**Mel-18 transcription levels for the clinical groups**. Boxplot showing the transcription level of Mel-18 within the clinical groups. Group legends as in figure 3.

The relative transcription level of Mel-18 in breast tissue from non cancer controls, fibroadenomas, tissue taken in the vicinity of the tumor and the tumor itself, is demonstrated in figure 3 and 4 and summarized in table 3. The mRNA transcription level of Mel-18 was statistically significantly higher in normal breast tissue from non cancer controls compared to normal tissue from cancer patients (p < 0.001).

### There was a inverse relationship between the transcription level of Mel-18 and Bmi-1

**Figure 5:**
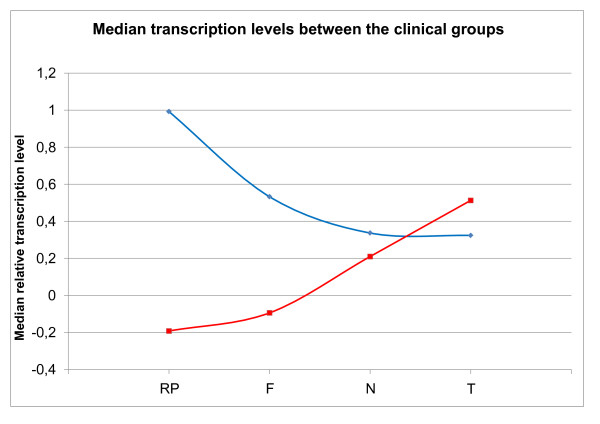
**Median transcription levels between the clinical groups**. Comparison of the median transcription level of Bmi-1 and Mel-18 for each clinical group shows an inverse correlation of the transcription level.

When we compared the transcription level of Bmi-1 and Mel-18 in all categories (NC, non cancer controls, F, fibroadenomas N, normal and T tumor) we observed an inverse relationship between the transcription level of the two genes (figure 5).

### Quantitative Real-time PCR

**Figure 6:**
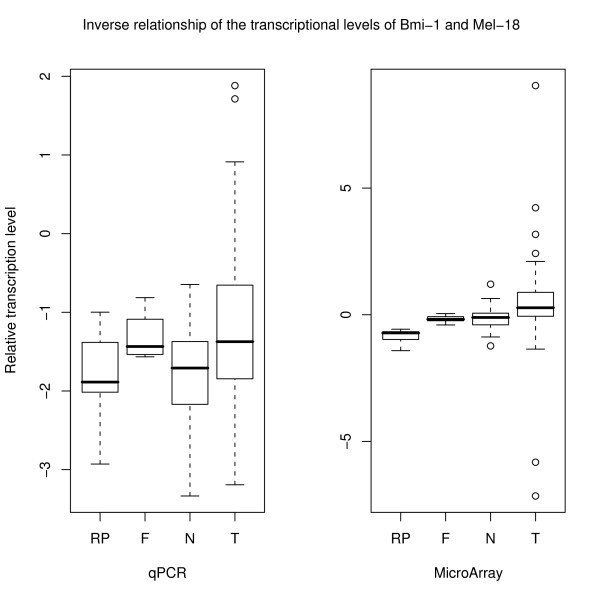
**Quantitative real-time PCR shows the same inverse relationship between the transcriptional levels of Bmi-1 and Mel-18 as the microarray data**. The qPCR results were calculated using the comparative C_T_-method (-ΔΔC_T_) while the microarray results are given as the difference in expression of the two genes (log_2_(expression of Bmi-1/expression of Mel-18)).

To validate the results obtained by the microarray analysis showing a clear inverse relationship between the transcription levels of the two genes, transcription levels of Bmi-1 and Mel-18 were also analyzed using qPCR. Some of the samples could not be validated due to insufficient mRNA amounts but all the groups were represented (9 non cancer controls, 4 fibroadenomas, 2 DCIS, 22 normals from cancer patients, and 69 tumors). Based on the work of McNeill et al [27] PPIA and MRPL19 were included as endogenous controls. However, analysis of our results showed large variations in transcription levels between the groups, especially for PPIA (results not shown). As an alternative approach, we therefore directly compared the transcriptional level of Bmi-1 to that of Mel-18 (figure 6).

### Results of immunohistochemistry

**Figure 7:**
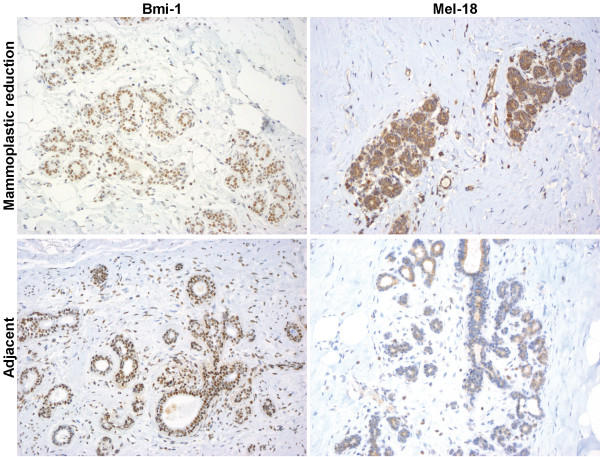
**Immunostaining**. Normal tissue from non cancer controls (upper panel) and normal tissue adjacent to a tumor (lower panel) of Bmi-1 (left) and Mel-18 (right). Demonstrating the increase in staining of Bmi-1 in tumor tissue, and the decrease in staining of Mel-18 in the same tissue. These are the outer limits and the staining of the groups in between are described in the text.

In our material all of the samples stained positive both for Bmi-1 and Mel-18 but with different intensity. When protein expression of Bmi-1 was evaluated using immunohistochemsitry, as illustrated in figure 7 we observed that almost for every case the expression intensity was stronger in normal tissue from breast cancer patients compared to normal breast tissue from non cancer patients, indicating differences in amount of protein in the cells between these two groups.

When evaluating the protein expression of Mel-18 using immunohistochemistry, breast tissue from non cancer individuals exhibited stronger expression intensity compared to normal tissue from cancer patients for almost every case, indicating probably higher amount of Mel-18 protein in breast tissue from non cancer controls.

However, both observations were subjectively made by a pathologist and no statistical differences were seen in numbers of positive cells between breast tissue from non cancer controls compared to normal tissue taken from a breast with a malignant tumor in the breast.

## Discussion

In the present study we have analyzed levels of Mel-18 and Bmi-1 in normal breast tissue samples from patients operated for cancer, and compared to breast tissue samples from patients operated for non malignant condition and with no previously history of malignant disease.

We have shown that level of Bmi-1 is different in the normal breast tissue from cancer patients compared to normal breast tissue from non cancer controls at the mRNA level and there is also differences in staining intensity at the protein level, indicating that gene alterations associated with tumor development is already detected in the normal tissue, leading to higher risk for development of a malignant disease in the breast. At the same time we observed that at the protein level both genes were expressed in all studied tissue types, with no statistical differences in the numbers of positive cells between breast tissue from non cancer controls compared to normal tissue taken from a breast with a malignant tumor in that same breast. This may suggest that it is the change of expression above a certain threshold that may matter in exerting a cancer phenotype.

As Bmi-1 and Mel-18 are involved in cell aging [6,17], we looked for confounding effects of age of the patient even though cell aging and aging of a person is not the same. Accounting for this we did regression analysis by ANOVA and found that age is not a significant factor in this respect (p = 0.47 for Bmi-1 and p = 0.61 for Mel-18). The different clinical groups on the other hand, were highly significant (p = 0.0013 for Bmi-1 and p = 0.013 for Mel-18).

The results from present study indicate that normal breast tissue in cancer patients carries different characteristics than that in women without previous history of malignant disease. One may speculate that it is possible to stratify women in risk groups for development of malignant tumor in the breast, according to transcription level ratios of genes like Bmi-1 and Mel -18.

After the introduction of mammography as a screening method in most countries, breast tumors are more often diagnosed at early stage. Most of the lesions detected by mammography are benign, where benign histology is confirmed by histological diagnosis of a biopsy. However, new cancer cases known as "interval cancers" still emerge and are diagnosed at later stage. The results from the present study indicate that it may be possible to distinguish between patients at risk by analyzing the normal breast tissue for genes like Bmi-1 and Mel-18.

When comparing transcription level of mRNA for Bmi-1 between different sources of tissue, we observed a highly statistical difference in levels of transcription level. In normal tissue from breast cancer patients Bmi-1 mRNA was up-regulated, compared to tissue from breast without history of malignant disease. Again, there was no correlation between mRNA transcription level and the amount of cells positive for Bmi-1 in different groups, although intensitivity of immunoreactions were different between different groups and followed the same pattern as mRNA transcriptional level, indicating that there may be "more" protein in cells where mRNA transcriptional level was found to be high.

However, as in the case of Mel-18, mRNA transcription level is of transcripts isolated from all cells from which total RNA was isolated together and cannot directly correlate to the number of positive cells stained by immunohistochemistry. The number of cells in both specimens (for mRNA and protein analysis) is unknown and cannot be equal. Nevertheless the intensity of the immunohistochemistry, indicating amount of protein per cell, was inversely to what was observed for Mel-18 being higher in almost all samples from breast cancer patients compared to controls. Bmi-1 expression is necessary for normal cell cycle dynamics. It is possible that there may be "threshold" of Bmi-1 protein expression, and when this "threshold" is overridden, this protein may start to function as an oncogene. The highest transcription level of Bmi-1 was observed in invasive tumor tissue. Expression of Mel-18 was also analyzed. When transcription level of Mel-18 was analyzed in normal tissue from breast cancer patients, tumor tissue and tissue from patients without malignant disease, the lowest transcription level was observed in tumor tissue, and the highest in tissue from benign breast tissue. If this correlation reflects a direct functional interaction between Bmi-1 and Mel-18 is not possible to evaluate in this study. Nevertheless, we believe that expression analyses of both proteins may be an important tool for stratifying patients at risk for development of malignant disease in the breast. This is before the onset of an eventual malignant disease and as Silva et al suggest [19] it can further be used as a diagnostic marker in patients who have already been diagnosed with breast cancer.

Data comprehensively comparing gene expression between histologically normal breast epithelium of breast cancer patients and cancer-free controls is limited. Similarly as our study Tripathi et al [28] did global gene expression of these two groups and conclude that cancer-related pathways are already perturbed in normal epithelium of breast cancer patients. This is cohesive to our study. Chen et al [29] also had the intention of studying malignancy-risk gene signature in histologically-normal breast tissue, but they compared the tumor tissue to the histologically normal tissue adjacent to the tumor, which is also part of what has been done in our study.

Saeki et al [30] reported a similar study on Bmi-1 where they found transcription level of the gene to be ten times higher in cancer tissue than non cancer controls. This is in coherence with our results except for the magnitude. A reason for this may be the observed large variation in tumor Bmi-1 levels in both materials and tumor heterogeneity may be responsible for the observed differences. By using laser micro dissection one could more accurately see the exact tissue from which mRNA was extracted.

## Conclusion

In summary; we have in the present study demonstrated for first time that the levels of Mel-18 and Bmi-1 is different in normal tissue from breast cancer patients compared to breast tissue from non cancer controls. The transcription level of Bmi-1 and Mel-18 in all clinical categories (non cancer controls, fibroadenomas, normal, and tumor) was inversely correlated. Mel- 18 and Bmi-1 are two essential proteins in stem cell renewal pathways. Results from the present study indicate that expression profile analyses of that Mel-18 and Bmi-1 may be a tool for stratifying women at risk for development of malignant disease.

## List of abbreviations

PcG: Polycomb Group of Proteins; DCIS: Ductal Carcinoma in Situ; UHR: Universal Human Reference; RP: Reduction Mammoplasty; F: Fibroadenoma; N: Normal; T: Tumor; and qPCR: Quantitative Real-time PCR;

## Competing interests

The authors declare that they have no competing interests.

## Authors' contributions

MR was involved in the design of the study, collected the clinical data, contributed to the tissue collection and immunohistochemical analysis and is responsible for the preparation of the manuscript. TL performed the microarray experiments, contributed to the microarray, qPCR and statistical analysis, and is responsible for the preparation of the figures and the final formatting of the manuscript. AJN performed the immunohistochemical analysis. HV contributed to the microarray analysis. VK and IB designed the study, contributed to the preparation of the manuscript and supervised the work of MR, TL and HV. All authors have read and approved the final manuscript.

## Pre-publication history

The pre-publication history for this paper can be accessed here:

http://www.biomedcentral.com/1471-2407/10/686/prepub
